# Phytochemical Profile, Antioxidant, Antimicrobial, and Antidiabetic Activities of *Ajuga iva* (L.)

**DOI:** 10.3390/life13051165

**Published:** 2023-05-11

**Authors:** Soukaina Saidi, Firdaous Remok, Nadia Handaq, Aziz Drioiche, Aman Allah Gourich, Naoual El Menyiy, Smail Amalich, Mohamed Elouardi, Hanane Touijer, Mohamed Bouhrim, Latifa Bouissane, Hiba-Allah Nafidi, Yousef A. Bin Jardan, Mohammed Bourhia, Touriya Zair

**Affiliations:** 1 Research Team of Bioactive Molecules and Environment Chemistry, Laboratory of Innovative Materials and Biotechnology of Natural Resources, Faculty of Sciences, Moulay Ismail University, Meknes 50070, Morocco; 2 Laboratory of Molecular Chemistry, Materials and Catalysis, Faculty of Science and Technologies, Sultan Moulay Slimane University, Beni Mellal 23000, Morocco; 3 Plant Valorization and Protection Research Team, Laboratory of Environmental Biology and Sustainable Development, Higher Normal School of Tetouan, Abdelmaek Essaadi University, Tetouan 93000, Morocco; 4 Laboratory of Pharmacology and Phytochemistry, National Agency of Medicinal and Aromatic Plants, Taounate 34025, Morocco; 5 Laboratory of Biological Engineering, Team of Functional and Pathological Biology, Faculty of Sciences and Technology Beni Mellal, University Sultan Moulay Slimane, Beni Mellal 23000, Morocco; 6 Department of Food Science, Faculty of Agricultural and Food Sciences, Laval University, Quebec City, QC G1V 0A6, Canada; 7 Department of Pharmaceutics, College of Pharmacy, King Saud University, Riyadh 11495, Saudi Arabia; 8 Department of Chemistry and Biochemistry, Faculty of Medicine and Pharmacy, Laayoune 70000, Morocco; bourhamohammed@gmail.com

**Keywords:** *Ajuga iva*, quality control, DPPH, FRAP, antimicrobial activity, OGTT toxicity, α-amylase

## Abstract

In Morocco, many applications in ethnomedicine on *Ajuga iva* (L.) have been recognized as able to treat various pathologies such as diabetes, stress, and microbial infections. The objective of this work is to carry out phytochemical, biological, and pharmacological investigations on the extracts of *Ajuga iva* leaves in order to confirm its therapeutic effects. The phytochemical screening carried out on the different extracts of *Ajuga iva* showed its richness in primary (lipids and proteins) and secondary metabolites (flavonoids, tannins, reducing compounds, oses, and holoside. The best contents of polyphenols, flavonoids, and tannins evaluated by spectrophotometric methods were found in the hydroethanolic extract (69.850 ± 2.783 mg EAG/g DE, 17.127 ± 0.474 mg EQ/g DE, 5.566 ± 0.000 mg EQC/g DE), respectively. Analysis of the chemical composition of the aqueous extract by LC/UV/MS revealed 32 polyphenolic compounds including ferulic acid (19.06%), quercetin (10.19%), coumaric acid (9.63%), and apigenin-7-(2-O-apiosylglucoside) (6.8%). The antioxidant activity of *Ajuga iva* extracts was evaluated by three methods (DPPH*, FRAP, CAT). The hydroethanolic extract recorded the strongest reducing power: DPPH* (IC_50_ = 59.92 ± 0.7 µg/mL), FRAP (EC_50_ = 196.85 ± 1.54 (µg/mL), and CAT (199.21 ± 0.37 mg EAG/gE). A strong correlation between phenolic compounds and antioxidant activities was confirmed by the determination of Pearson’s coefficient. The antimicrobial activity of *Ajuga iva* studied by the microtiter method revealed potent antifungal and antibacterial qualities against *Candida parapsilosis* and *Staphylococcus aureus* BLACT. An in vivo oral glucose tolerance test (OGTT) using normal rats revealed that the antihyperglycemic action of the aqueous extract significantly reduced postprandial hyperglycaemia at (30 min, *p* < 0.01) and area under the curve (AUC glucose), *p* < 0.01. Similarly, the aqueous extract, tested on pancreatic α-amylase enzyme activity in vitro and in vivo significantly inhibited pancreatic α-amylase activity with IC_50_ = 1.52 ± 0.03 mg/mL. In conclusion, the extract from *Ajuga iva* could be a good source of bioactive molecules, which exhibit potent antioxidant and antimicrobial activity, as well as strong antidiabetic activity, for applications in the pharmaceutical industry.

## 1. Introduction

Medicinal plants contain a variety of bioactive compounds that have been used for thousands of years as traditional therapies for a wide range of human ailments. Nearly 80% of people in underdeveloped nations use medicinal plants as their primary source of healthcare according to the World Health Organization [1]. In fact, oxidative stress has been associated with a number of human diseases, including diabetes, Alzheimer’s, Parkinson’s, cancer, and cardiovascular diseases [2,3,4]. Reactive oxygen species (ROS) generation and the body’s antioxidant defense capabilities are out of equilibrium, which leads to oxidative stress [5]. Therefore, the cytotoxic control of ROS is generally attributed to antioxidant systems [2]. Natural antioxidants, such as flavonoids, phenolics and tannins, protect against diseases associated with oxidative stress [6]. A decrease in antioxidants may also cause the emergence of diabetic problems [7]. Persistent hyperglycemia is a hallmark of the metabolic syndrome known as diabetes, which develops when the pancreas either produces insufficient insulin (insulin secretion insufficiency) or when the body no longer effectively uses insulin (insulin resistance) [8,9]. Additionally, more than 90% of diabetic people have type 2 diabetes mellitus, a complex illness [10]. This type of diabetes stems from insulin resistance, which is strongly associated with several triggering factors, including poor diet, genetic predisposition, and lack of physical activity [10,11]. Between 5% and 10% of all diabetics have type 1 diabetes, often known as “juvenile” diabetes, which is caused by the pancreas’ failure to release insulin as a result of certain genetic and environmental variables [8,9,10]. Modern medicine has played a crucial role in the development of anti-diabetic drugs, which have significantly improved the management of diabetes. These drugs significantly reduce the postprandial rise in plasma glucose and insulin levels in diabetic patients by inhibiting the activity of α-glucosidases [12]. Inhibitors of α-glucosidase are often based on mimetic conformation of the substrate and/or the positive charge of transition state [13]. The inhibitors of α-glucosidase that are most involved in the digestion of carbohydrates are iminosugars such as 1-deoxynojirimycin (DNJ), castanospermine, swainsonine, fagomine, etc., [13,14]. However, it has recently been shown that these iminosugars, along with medications such as metformin and acarbose, have many drawbacks, such as nausea, bloating, weakness, etc. [11,15,16].

Therefore, attention has been directed towards the use of medicinal plants to identify and isolate natural bioactive molecules endowed with substantial antidiabetic effects with less harmful impact on human health. One of those potent prospective medicinal plants is *Ajuga iva* (L.), which has a long history of usage in the conventional medical system as an anti-diabetic, an antioxidant, and an antibacterial. The Lamiaceae family includes the herbaceous plant species *A. iva* (L.), which is extensively distributed in southern Europe, Egypt, Tunisia, and Morocco [17]. This plant species has a number of slang names, including Chendghoura and Toutoulba [18]. Some *Ajuga* species are regarded as weeds, yet many are employed in horticulture as ground or border cover and in rock gardens [19]. In Morocco, many ethno-medical uses have been reported for this plant. The aerial portion (leaves and flowers) of *A. iva* has been used as a decoction to treat a variety of conditions, including kidney and digestive disorders, diabetes, hypertension, painful menstruation, rheumatic pain, eye infections, cardiovascular disorders, allergies, cancer, and rheumatism [20,21,22,23,24,25,26,27]. Recently, several of its ethno-medical uses have been validated by studies by experimental scientists in laboratories, in vivo and in vitro demonstrating that the extracts of *A. iva* possess significant antibacterial, anticancer, antioxidant, antidiabetic, insecticidal, antiviral, anticancer, analgesic, and antihypercholesterolemic properties [28,29,30,31,32]. In addition, phytochemical investigations on *A. iva* revealed the presence of a wide spectrum of chemical constituents belonging to flavonoids, tannins, terpenoids, steroids, fatty acids, and phenolic acids (caffeic acid and chlorogenic acid, etc.) and other principles, such as ajugarine C_24_H_34_O_7_ (MW: 392) [23,33,34]. In addition, *A. iva* includes essential oils, as well as anthocyanins, neoclerodane diterpenoids, and other compounds [35]. Therefore, the pharmacological and biological activities of *A. iva* are mainly explained by all the primary and secondary metabolites present in its leaves and fruits [21]. The purpose of this study was to corroborate the traditional use of *A. iva* (L.) through comparative phytochemical characterizations of phenolic compounds in its extracts and evaluation of their antioxidant, antimicrobial, and antidiabetic effects in vitro. In addition, the antihyperglycemic activities, the inhibition of α-amylase, as well as the toxicity of the decoction of *A. iva* were performed in vivo.

## 2. Materials and Methods

### 2.1. Chemicals Products

The majority of the chemicals and solvents were bought from Sigma Aldrich (St. Louis, MO, USA). Pancreatic α-amylase and other reagents were acquired from Sigma-Aldrich, whereas acarbose was bought from Bayer Schering Pharma (Berlin, Germany).

### 2.2. Plant Material

**Figure 1:**
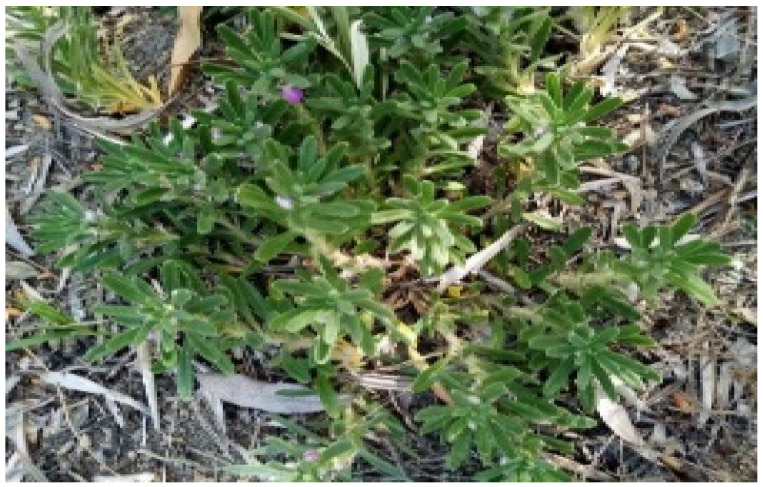
*Ajuga iva* (L.) (Soukaina Saidi and Touriya Zair 2021).

*Ajuga iva* leaves were gathered in June, 2021 from the Masmouda region of Morocco (X = 471170 W; Y = 464816 N; Z = 320 m). The leaves were then air-dried at room temperature in the shade before being ground into a powder to create the extracts. The Scientific Institute of Rabat, Morocco, carried out the plant’s botanical identification. Table 1 and Figure 1 contain comprehensive data on this species [36].

### 2.3. Microbial Material

Nine bacterial strains and eight fungus strains were used to test the antibacterial activity of *A. iva* L. extracts (Table 2). All strains were cultivated in subcultures after being revived in Mueller Hinton and Sabouraud broths (20% glycerol stock) at 80 °C.

### 2.4. Animals

The Wistar rats weighing 200–250 g and the Swiss albino mice weighing 25–30 g used in this study were purchased from the animal house of the Department of Pharmacology of the National Agency of Medicinal and Aromatic Plants (Taounate, Morocco). The animals were housed under controlled laboratory conditions of temperature (22 ± 2 °C) and light (12 h light/dark cycles). The animals were housed in breeding-friendly environments with unrestricted access to food and water.

### 2.5. Preparation of Extracts

#### 2.5.1. Preparation of the Decocted Extract

In a 600 mL batch of distilled water, 30 g of *A. iva* (L.) leaf powder was cooked for 60 min at 80 °C with some stirring. The solution was filtered after cooling, and then it was forced to evaporate. The resulting residue was subsequently dried at 60 °C. The powder obtained represents the decocted extract of leaves of *A. iva* (L.) (Ed).

#### 2.5.2. Preparation of Aqueous and Hydroethanolic Extracts

A total of 30 g of plant material from the leaves of *A. iva* (L.) was extracted with 600 mL of distilled water (aqueous extract) or 600 mL of 70/30% ethanol–water (hydroethanolic extract) using a Soxhlet type apparatus for 12 h. In a vented oven set at 60 °C, the filtrates were dried. In sterile vials, the dried crude extracts of the various aqueous (EAq) and hydro-ethanolic (Eeth) solvents were gathered and kept at room temperature until use.

### 2.6. Parameters Evaluated of Plant Material

#### 2.6.1. Water Content

The procedure was carried out in accordance with the AFNOR standard (NF-V03-402, 1985) [37]. The protocol consists of putting 5 g of the sample in a ventilated oven at 103 °C for 24 h until constant weight. The test was conducted three times, and the average was calculated. The water content was measured after drying the sample by the following formula (Equation (1)).

(1) $$WC\%=(\frac{M_{0}-M_{1}}{M_{0}})\times 100$$

where *WC*: Water content (%); *M*_0_: Mass before drying (g); *M*_1_: Mass after drying (g).

#### 2.6.2. Ashes

The ash content of *A. iva* (L.) leaves was determined in accordance with the standard (NFV05-113, 1972) [38]. A total of 5 g of the vegetable powder was weighed into preheated porcelain crucibles. The method’s basic idea is to calcine the sample at 550 °C in a muffle furnace until white ashes with a consistent mass are produced. The average was calculated after the test was administered three times. The following formula determines the organic matter content:

(2) $$OM\%=(\frac{W_{1}-W_{2}}{TS})\times 100$$

where *OM*%: Organic material; *W*_1_: Capsule and sample weight before calcination; *W*_2_: Capsule and sample weight after calcination; *TS*: Test sample.

Ash content is determined using the formula below:

Ash% = 100 − *OM*%.

#### 2.6.3. pH Determination

The principle consists of bringing the quantity of vegetable matter into contact with the quantity of distilled water (ratio 1:5). After filtering, the mixture was allowed to cool. A pH meter with an electrode was used to measure the pH [39].

#### 2.6.4. Titratable Acidity (TA)

A total of 10 g of the vegetable powder was brought into contact with 100 mL of boiling distilled water; the whole extract was stirred for 15 min, then filtered. A total of 10 mL of the filtrate and 20 mL of water were placed in a 100 mL beaker; then, a few drops of phenophthalein were added and the titration was carried out with a solution of NaOH (0.01 N) until a persistent pink color was obtained; the volume of NaOH used was noted. This volume was converted into equivalent citric acid, and the titratable acidity was determined in accordance with the following relationship [40].

(3) $$TA=\frac{weight\;of\;acid\;equiv.\;\times \;normality\;of\;NaOH\;\times \;vol.of\;titration\left(mL\right)\times Dilution\;factor}{sample\;weight\;in\;(g)}$$

#### 2.6.5. Heavy Metals Assessment Using ICP-AES

Inductively coupled plasma atomic emission spectrometry (ICP-AES) (Ultima 2 Jobin Yvon, HORIBA Jobin Yvon, Stow, MA, USA) was used to determine the presence of all analyzed elements, including arsenic (As), cadmium (Cd), chromium (Cr), iron (Fe), lead (Pb), antimony (Sb), and titanium (Ti). The study used aqua regia and a mineralization technique established by (AFNOR 1999) to determine concentrations of key elements in plant material. The sample was prepared by combining 0.1 g of crushed plant material with 3 mL of aqua regia, consisting of 1 mL of nitric acid (HNO_3_; 99%) and 2 mL of hydrochloric acid (HCl; 37%), which was then refluxed at 200 °C for two hours. Then, it was filtered through a 0.45 μm membrane and was complemented by 15 mL of distilled water [41].

### 2.7. LC/UV/MS Analysis of Decoction Extracts

The LC-UV-MS analysis was conducted with a PLATIN LC/HPLC system in conjunction with a UV detector. The mobile phase consists of a mixture of Solvent A (acetonitrile + formic acid 0.1%) and Solvent B (water + formic acid 0.1%). The elution gradients used were as follows: 0–5% B for 1 min, followed by 5–20% B for 0.5 min, 20% B for 3.5 min, 20–100% B for 4 min, 100% B for 2 min, and finally, re-equilibration at 100–0% B for 0.5 min, before returning to 0% B for 2.5 min. The system needed 70% methanol for cleaning. The decoction was diluted in a 1:1 methanol/water solution to reach a concentration of 1 mg/mL. After that, a 2 mm PTFE filter was used to filter it. In each assay, 0.004 mL of the decocted extract was injected. The temperature was adjusted to 30 °C and the flow rate to 0.3 mL/min. Based on bibliographic data, a few standards were picked for this inquiry. They were injected in the exact same conditions as a decoction. Cinnamic acid, luteolin, apigenin, myricetin, ferulic acid, quercetin, vanillic acid, chlorogenic acid, ascorbic acid, caffeic acid, rosmarinic acid, protocatechuic acid, gallic acid, and coumarin are some of the standards in this group [42].

### 2.8. Phytochemical Study of Ajuga iva *(L.)* Leaves Extracts

#### 2.8.1. Phytochemical Screening

The main chemical groups of the extracts under investigation are sought after by phytochemical screening, a qualitative examination. This method involves detecting a change in color or precipitation that occurs when a specific reagent is added. All experiments were conducted in accordance with the widely used phytochemical techniques, as reported by Dey and Harborne (1989) and Gibbs Darnley (1974) [43,44]. Several reagents were used for the qualitative analyses. The flavonoids were identified by the cyanidin reaction. Tannins were characterized by reaction with ferric chloride. The Stiasny response emphasized the difference between the two classes of tannins. Alkaloids were characterized using Dragendorff’s and Mayer’s reagents. The Fehling solution was used to identify the presence of reducing compounds. When the solution was agitated, the formation of foam was used to indicate the presence of saponins. The Liebermann–Buchard reaction was used to identify terpenes and sterols. The results were graded as follows: +++ for strong positive result, ++ for moderately positive result, + for weakly positive result, and − for negative result.

#### 2.8.2. Dosage of Phenolic Compounds

##### Determination of Total Polyphenol Content (TPC)

Total phenol content (TPC) was measured in accordance with Singleton et al. (1965) with slight modifications [45]. Into a volumetric flask of 50 mL, an aliquot (20 μL) of each extract of *A. iva* was mixed with 1.5 mL of Folin–Ciocalteu reagent (10%) and 1.5 mL of sodium carbonate (Na_2_CO_3_). After that, the mixture was stirred and adjusted with distilled water till the gauge line. The absorbance was measured at 760 nm using UV–VIS spectrophotometer after an incubation period of 1 h in the dark. A calibration curve of gallic acid was prepared, and the results were expressed as milligrams of gallic acid equivalents per gram of extract (mg GAE/gE) by using the following formula (Equation (4)):

(4) $$TPC=\frac{C\times V_{0}}{m_{\begin{array}{c} extract \end{array}}}\times D$$

where *TPC*: Total phenolic compounds; C: Concentration assessed based on the calibration curve; *V*_0_: Volume of the entire extract; *m_extract_*: Mass of extract.

$$D=\frac{V_{f}}{V_{i}}$$

where D: Dilution factor; *V_f_*: Final volume to be measured in a spectrophotometer; *V_i_*: Volume taken from the extract to be tested.

##### Determination of Flavonoid Content (FC)

Total flavonoid content (FC) was determined using a colorimetric method of AlCl_3_ [46]. A volume of 10 μL of each extract of *A. iva* was mixed with 10 µL of AlCl_3_ 10% (*w*/*v*) and 2 mL of distilled water. The mixture was made up to 5 mL with pure methanol. After incubation for 30 min at room temperature in the dark, the absorbance was measured at 433 nm using a UV–VIS Spectrophotometer. The results were expressed as milligrams of quercetin equivalents per gram of extract (mg QE/gE) by using the following formula Equation (5):

(5) $$TFC=\frac{C\times V_{0}}{m_{\begin{array}{c} extract \end{array}}}\times D$$

where *TFC*: Total flavonoid compounds; C: Concentration assessed based on the calibration curve; *m_extract_*: masse of extract.

##### Determination of Condensed Tannin Content (CT)

The condensed tannin content (CT) was quantified in accordance with the Vanillin-HCl method [47]. An aliquot (20 μL) of each extract of *A. iva* was mixed with 3 mL of vanillin solution (4%) (prepared in methanol) and 1.5 mL of concentrated hydrochloric acid (37%). Then, the mixture was incubated at room temperature for 20 min and the absorbance was measured at 499 nm. The condensed tannin content of the extracts was expressed as milligrams catechin equivalents (mg CE/g CE) per gram of extract using Equation (6).

(6) $$TCT=\frac{C\times V_{0}}{m_{\begin{array}{c} extract \end{array}}}\times D$$

where *TCT*: Total condensed tannin; C: Concentration assessed based on the calibration curve; *V*_0_: Volume of the overall extract; *m_extract_*: masse of extract.

### 2.9. Evaluation of Antioxidant Power

Three distinct techniques, DPPH, FRAP, and CAT, were used to assess the antioxidant strength of the *Ajuga iva* (L.) extracts.

#### 2.9.1. Radical Scavenging Activity (DPPH*)

The free radical scavenging activity of *A. iva* extracts, based on the scavenging activity of the (2.2-diphenyl 1-picryl hydrazyl) DPPH* free radical, was carried out using Wong et al. (2006) method [48] with some modifications. A 200 μL aliquot of the different concentrations of extracts and standards (ascorbic acid) were added to 2.8 mL of freshly DPPH* ethanol solution (0.024 mg/mL). After an incubation period of 30 min in the dark, the absorbance was measured at 515 nm using a UV-VIS spectrophotometer (V-1200). Ethanol was used as a negative control. The percentage of DPPH* inhibition was evaluated using Equation (7).

(7) $$PI\%=\frac{A_{0}-A}{A_{0}}\times 100$$

where PI: Inhibition of DPPH (%); $A_{0}:$ Absorbance of negative control; A: Absorbance of the extract/standard.

#### 2.9.2. Ferric Reducing Antioxidant Power (FRAP)

The FRAP, ferric reducing antioxidant power, of the extracts was determined by evaluating the ability of the extract to reduce a FeCl_3_ solution as described by Oyaizu (1986) [49].

An aliquot of 0.5 mL of extract was mixed with 2.5 mL of 0.2 M sodium phosphate buffer (pH 6.6) and 2.5 mL of 1% potassium ferricyanide. The mixture was incubated at 50 °C for 20 min, and then 2.5 mL of 10% trichloroacetic acid was added. This mixture was centrifuged at 3000 rpm for 10 min. A total of 2.5 mL of the supernatant was mixed with 2.5 mL of water and 0.5 mL of 0.1% ferric chloride solution. The absorbance was measured at 700 nm by a spectrophotometer (V-1200), and then the ferric reducing ability was calculated using ascorbic acid as a standard. The increased absorbance of the reaction mixture indicated increased reducing power.

#### 2.9.3. Total Antioxidant Activity (CAT)

The total antioxidant capacity (CAT) was determined in accordance with the method described by Prieto et al. (1999) [50]. A total of 5 µL of each extract of *A. iva* was combined with 3 mL of reagent solution (0.6 M sulfuric acid, 28 mM sodium phosphate, and 4 mM ammonium molybdate). The mixture was incubated at 95 °C for 90 min. Then, the absorbance of the solution was measured at 695 nm using a spectrophotometer (V-1200) against blank after cooling to room temperature. The antioxidant activity was expressed as milligram equivalents of ascorbic acid per gram of extract (mg EAA/g E).

### 2.10. Antimicrobial Activity

To determine the minimum inhibitory concentration (MIC) of each *A. iva* extract, a sterile 96-well microtiter plate was employed, along with resazurin as a cell growth indicator [51,52,53,54]. Each well of a 96-well microtitration plate was filled with 100 µL of Mueller Hinton broth for bacteria or Sabouraud broth for fungi [55]. The first well on the plate was filled with 100 µL of extract (200 mg/mL) prepared into (30/70)% ethanol/distilled water, which had been diluted serially by 1/2, 1/4, 1/8, 1/16, 1/32, and 1/64 before being added. Subsequently, 100 μL of inoculum was introduced into each well, and the microtiter plate was incubated for 24–48 h at 37 °C. After introducing 10 μL (6.75 mg/mL) of resazurin solution to each well, the samples were incubated for 2 h at 37 °C, and the MIC was determined. The reduction in resazurin and the subsequent bacterial or fungal growth are shown by a shift from blue to pink. The MIC value was calculated by obtaining the extract at the lowest concentration at which the color change occurred. In order to determine the minimum bactericidal or fungicidal concentration (MBC or MFC), a volume of 10 μL was extracted from wells where no visible growth was observed, and it was subsequently inoculated on Mueller–Hinton agar plates for bacterial growth or Sabouraud agar for fungal growth. The plates were then incubated for 24 h at 37 °C. Following incubation, MBC/MFC is found to be the lowest concentration that prevents colony formation on solid agar medium [56,57].

### 2.11. Acute Toxicity of Decoction of Ajuga iva *(L.)*

The objective of the toxicity test was to demonstrate that therapeutic doses of *A. iva* decoction in normal mice do not cause any short-term harm. In reality, five groups of albino mice (20–35 g) were randomly selected from two batches and divided into five groups (*n* = 6; ♂/♀ = 1). The treated groups were administered 0.5 g/kg, 1 g/kg or 2 g/kg of *A. iva* decoction, while the control group received distilled water (10 mL/kg). Before starting the experiment, the mice were weighed and then rapidly received the extract orally. Subsequently, the mice were continuously monitored for 10 h to identify any signs of toxicity. Mice were observed daily for 14 days to detect any novel clinical or behavioral signs of damage. The experimentation followed the guidelines recommended by the Organization for Economic Co-operation and Development (OECD) [58].

### 2.12. Antihyperglycemic Effect of Decoction of Ajuga iva *(L.)*

The hypoglycemic effect (post-prandial blood glucose) of *A. iva* (L.) decoction in vivo was discovered using an oral glucose tolerance test [59]. Three groups of normal rats were formed (*n* = 6; ♂/♀ = 1). The control group, consisting of normal rats, was administered distilled water at a dose of 10 mL/kg. The treatment groups were given decoction extract (400 mg/kg) and glibenclamide (2 mg/kg). The oral glucose tolerance test was conducted as follows: glycaemia was measured at time zero, immediately following the administration of the test substance to the rats (decoction or glibenclamide). Another blood glucose reading was performed 30 min after the animals had a 2 mg/kg D-glucose excess. The fluctuation in blood glucose was then observed for 2 h.

### 2.13. Pancreatic α-Amylase Inhibitory Effect of Decoction of Ajuga iva *(L.)*

#### 2.13.1. In Vitro Test

The method published by Boulfia et al. [60] was used with minor changes to test the decoction’s inhibitory effect on the enzymatic activity of pancreatic α-amylase. In a test tube, an aliquot of a 200 µL extract or standard at different concentrations (0.18–2.11 mg/mL) was mixed with 200 μL of the phosphate buffer solution (0.02 M; pH = 6.9). The pancreatic α-amylase enzyme solution (61.33 IU) was then diluted to a volume of 200 μL and added to each tube. The mixtures were then pre-incubated at 37 °C for 10 min. After this, 200 μL of starch solution (0.5%) was added, and the mixture re-incubated at 37 °C for 15 min. After stopping the enzymatic reaction by adding 600 µL of 2.5% DNSA to the tubes, they were incubated in a boiling water bath for 8 min. This reaction was then halted by heat shock, placing the tubes in an ice water bath, then adding 10 mL of distilled water to each tube. The absorbance at 540 nm was measured with a spectrophotometer (V-1200). Acarbose was used as a positive control. The inhibition rate for each extract and acarbose was determined by Equation (8). The alpha-amylase inhibitory activity was estimated using the following formula and reported as a percent inhibition

(8) $$\mathrm{Alpha-amylase inhibitory activity}\left(\%\right)=100\times [\frac{\left(A_{1}-A_{2}\right)-\left(A_{3}-A_{4}\right)}{A_{1}-A_{2}}]$$

where $A_{1}$: Control absorbance (enzyme + buffer); $A_{2}$: Absorbance of the control blank (buffer without enzyme); $A_{3}$: Sample absorbance (enzyme + extract); $A_{4}$: Absorbance of sample blank (extract without enzyme).

#### 2.13.2. In Vivo Test

To confirm the inhibitory effect of the *A. iva* (L.) decoction, an in vivo test was conducted on normal rats, considering the impact of the intestinal lumen on the decoction’s inhibitory properties. Three groups of normal rats (180–250 g) were formed after a 14 h fast (*n* = 6; ♂/♀ = 1). The treated groups either received the decocted extract (400 mg/kg) or acarbose (10 mg/kg), whereas the control group received distilled water (10 mL/kg). The oral starch tolerance test was carried out as follows: the product to be tested (distilled water, decoction or Acarbose) was administered (t = 0 min), 30 min later, a blood glucose measurement was performed, just after, a surplus of starch (3 g/kg) was given to the rats. After that, the blood glucose variation was assessed after 30, 60, and 120 min [56].

### 2.14. Statistical Analysis

Following a one-way analysis of variance (ANOVA) with Tukey’s post hoc test, the resulting data were reported in the form of means and standard deviations. Statistical significance was determined with criteria of *p* < 0.05, *p* < 0.01, and *p* < 0.001. R version 4.2.2 (31 October 2022) was used to generate heat maps and Pearson correlations between the biological activity of phenolic compounds and their chemical composition.

## 3. Results and Discussion

### 3.1. Quality Control for Ajuga iva *(L.)* Plant

The quality control of the plant material of *A. iva* (L.) was carried out, in accordance with the standards cited in supra, by determining the moisture content (MC), the pH, the titratable acidity, the ashes, and the ICP; the results obtained are shown in Table 3 and Table 4 below.

#### 3.1.1. Water Content

The moisture content of the raw powder of *A. iva* (L.) leaves measured by the oven method is about 11.56 ± 0.164%. This humidity level is linked to the water activity, so the drying of the plants is carried out to ensure good preservation of the samples by inhibiting the enzymatic activity, which prevents the degradation of certain components and the bacterial proliferation [61]. According to the criteria given by the European Pharmacopoeia in 2000, this content must not exceed 10%. The results of this analysis showed that the moisture content of *A. iva* (L.) was slightly above 10%. Moisture content slightly above 10% can improve the medium-term storage of our powder. This water content that was obtained was considered to be higher than the water content of 6.43%, which obtained from the leaves of *A. remota* Bens in Ethiopia [62]. Several factors contribute to this variability, the most important of which are climate, soil, harvest date, and drying and storage methods.

#### 3.1.2. pH Determination

The hydrogen potential tells us about the assimilability and good availability of nutrients by plants. The measured pH of *A. iva* (L.) is around 5.28 ± 0.098; this value is in the interval (4.0–6.5), placing our plant in the category of acidophilic plants without total limestone. The value found proves that this species has good absorption property of nutrients such as iron, which is well confirmed by the results of ICP analysis.

#### 3.1.3. Titratable Acidity

The titratable acidity value found in *A. iva* (L.) powder is equal to (37.866 ± 0.0075)% (Table 3). The titratable acidity indicates the quantity of free organic anions in the solution tested. According to Soro and coworkers, pH is inversely proportional to titratable acidity [63].

#### 3.1.4. Ash Content

Ash is a sort of mineral compound that, unlike organic materials, does not become volatile at high temperatures, hence their percentage tells us about the amount of these compounds. *A. iva* (L.) records a high ash content of around (16.3005 ± 0.2763)%. The rate recorded is slightly higher than those obtained at *A. iva* (L.) in Tunisia (13.0%) [64] and in Algeria (11.10%) [65]. Additionally, this rate is much lower than that found in *A. remota* Benth from Ethiopia (27.10%) [62]. According to Ammar and his collaborators [66], the ash content of the leaves of *A. iva* (L.) varies from one region to another; indeed, they found that for the two localities of Tunisia, Mograne and Nabeul, the contents varied between 15.2% and 24.3%. These differences between localities could be due to some external factors, such as climatic, soil, and environmental conditions. The content found makes our plant an important source of minerals for human nutrition.

### 3.2. Quantification of Metals

Determination of the content of minerals, including microelements iron (Fe), copper (Cu), and heavy metals cadmium (Cd), lead (Pb), antimony (Sb), chromium (Cr), arsenic (As), and titanium (Ti), was carried out in the leaves of *A. iva* (L.), the results of which are presented in Table 4. The results obtained indicate that iron was the most abundant microelement with contents of 2.3433 mg/g. For copper, a low concentration was noticed in the order of 0.0082 mg/g. As for heavy metals, considerable levels were noted as follows:

Cr (0.0297) > Pb (0.0243) > Sb (0.0239) > As (0.0213) > Cd (0.0015).

Thus, the leaves of *A. iva* (L.) are characterized by their richness in minerals, which may be responsible for their biological activity. Senhaji Souad and co-workers [67] reported that the metal analysis result in *A. iva* (L.)*. pseudova* (DC.) Briq collected in the region of Taza (Morocco) and showed an abundance of iron in comparison to the other elements measured. This comparison of results agrees with ours, where we found the iron concentration to be the highest in our sample.

Note that iron (Fe) is a necessary element for both humans and animals, as well as a necessary part of myoglobin and hemoglobin. Anemia can result from iron deficiency, which also causes gastrointestinal infections, nosebleeds, and cardiac infarctions. Reactive oxygen species, which Fe can produce, play a role in the pathophysiology of diabetes and its side effects, such as diabetic nephropathy. Thus, the use of a decoction of *Ajuga iva* (L.) provides a value of 234.33 mg of iron per 100 g of plant, which is pharmacologically significant in the absence of dietary iron [68,69].

For Copper (Cu), it is a component of several enzymes that plays a role in the synthesis of collagen and the regulation of cardiovascular function. However, its severe and chronic deficiency promotes anemia and causes cardiovascular changes and skeletal weakness. The estimated dietary and adequate intake for Cu^2+^ is only 0.9 mg. Our results record a content of 0.82 mg/100 g of *Ajuga iva* (L.), which is physiologically useful [68].

Lead (Pb) is a chemical that is frequently dangerous and is known to have a variety of toxic effects in living things, including those that are morphological, physiological, and biochemical in nature [70,71]. The temporary tolerable weekly intake for adults has been set by the FAO/WHO committee at 3 mg per person. Our findings revealed a physiologically acceptable Pb concentration of 2.43 mg/100 g of *Ajuga iva* [72].

Chromium (Cr) is an essential mineral element, in minute amounts in humans, for sugar and lipid metabolism, used as a glucose tolerance factor (GTF), and its deficiency can cause a disease called Cr deficiency, while its hexavalent form is extremely toxic and carcinogenic. In fact, the Recommended Dietary Allowance (RDA) reported in a review that a dose of 150–250 μg/day improved the diabetic symptoms of people with diabetes. Thus, chromium supplements can help treat glucose intolerance and type 2 diabetes [68,69]. It should be noted that 5.22 g of *A. iva* (L.) can yield 155 µg/d of Cr; this quantity is adequate for diabetic people. As a result, this plant can be thought of as a successful treatment for type 2 diabetes and other conditions brought on by chromium deficiency.

Cadmium (Cd) is a non-essential metal mainly stored in the liver and kidneys; it induces renal, cerebral, cardiac dysfunction, and lung and reproductive disorders. It has been officially listed as a rat and human lung carcinogen by the International Agency for Research on Cancer [69,70,71,72,73]. The FAO/WHO committee has proposed adopting a temporary tolerable weekly intake of 400 to 500 µg per person [72]. It should be mentioned that taking 450 µg of Cd weekly, obtained from 300 g of *A. iva* (L.) is within the limits of the value prescribed by FAO/WHO.

Arsenic (As) is a poisonous metalloid and one of the most significant environmental contaminants in the world. Inorganic arsenic is particularly harmful because it can have both short-term and long-term negative health consequences. According to recent reports, arsenic is a common environmental carcinogen that has been related to diabetes, cardiovascular disease, skin cancer, and bladder cancer. The results obtained for *A. iva* (L.) recorded a content of As of approximately 21.3 µg/g, which is within the range authorized for arsenic 1–30 µg/g by FAO/WHO [69,74,75,76].

Antimony (Sb) is a very rare element, often found in sulphides and sulfur salts. It exhibits a biochemical behavior comparable to that of arsenic and bismuth. Antimony’s toxicity is poorly understood, but Sb(III) species are often more toxic than Sb(V) species [77,78,79]. In a review prepared by Boland and collaborators, they reported that plants could be good potential phytoremediators for the remediation of metals, including Sb, and that the appropriate concentration of Sb in plant pytoremediators ranged from 3.92 to 143.69 mg/kg [80]. Our results recorded a concentration of the order of 23.9 mg/kg, which is included in the range of suitable and non-toxic concentrations.

### 3.3. Phytochemical Screening

The primary and secondary metabolites detected in *A. iva* (L.) leaf extracts are shown in Table 5. The results of these tests show that *A. iva* (L.) leaves are rich in primary metabolites. Indeed, *A. iva* (L.) contains a large amount of lipids followed by proteins and traces of reducing sugars and glycogen. With regard to secondary metabolites, *A. iva* (L.) is characterized by the presence of reducing compounds, oses, and holosides in large quantities. Additionally, gallic and catechin tannins, flavones, leucoanthocyanins, anthocyanins, sterols, and triterpenes are present in the leaves of this plant. Alkaloids are present in the form of traces, while mucilages and saponosides are totally absent in the leaves of this plant. A study by Senhaji and his collaborators also showed that *A. iva* (L.) is rich in catechic tannins, saponins, sterols, and flavonoids [67].

### 3.4. Determination of Extraction Yields and Contents of Total Polyphenols, Flavonoids, and Catechin Tannins

The results of the extraction yield and the contents of polyphenols, flavonoids, and catechin tannins of *A. iva* (L.) depending on the extraction methods used, are indicated in Table 6. We find that the extraction by decoction gives the greatest yield (17.016 ± 0.032)%, compared to the extraction by Soxhlet, whose aqueous extract has the value (11.048 ± 0.511)%, while the hydroethanolic extract records the value of (7.385 ± 0.662)%. These results agree with several similar studies, which showed that aqueous extracts showed higher yields than extracts obtained by organic solvents [81,82,83]. For example, extraction yields determined from extracts of *A. iva* (L.) obtained by acetone, ethanol, and water were respectively around 2.3%, 2.6%, and 24% [19]. Additionally, the extraction yield seems to be influenced by the degree of polarity of the solvent, the extraction method, the extraction time, as well as by the degree of polarity of the various components of the extract, such as the phenolic constituents [34]. With regard to the polyphenol and flavonoid contents, we note that the hydro-ethanolic extract presents (69,850 ± 2783) mg EAG/gE; (17.127 ± 0.474) mg EQ/gE respectively; these values are much higher than those evaluated in the case of the aqueous extract (53.148 ± 2.509) mg EAG/g E; (10.281 ± 0.521) mg EQ/gE, respectively, and the discount (40.786 ± 2.957) mg EAG/g E; (8.960 ± 0.370) mg EQ/g DE, respectively. As for the catechic tannin content, the three extracts (decocted, hydro-ethanolic, and aqueous) seem to have similar quantities, respectively, (5.746 ± 0.413) mg EQC/gE; (5.566 ± 0.000) mg EQC/gE; (5.792 ± 0.064) mg EQC/gE. These results agree with a study carried out by Saad and his collaborators [84]; they found that the methanolic extract is richer in polyphenols and flavonoids compared to the aqueous extract. This difference could be justified by the degree of polarity of each solvent to drive the phenolic compounds [84]. These phenolic compounds are secondary metabolites that have been extensively researched in a variety of medicinal plants, fruits, and vegetables [42,85]. They have antioxidant, antibacterial, and carbohydrase inhibitor properties. Additionally, flavonoids are a subclass of polyphenolic chemicals with a benzo-γ-pyrone structure that has shown to be effective against microorganisms [86].

### 3.5. Identification of the Chemical Composition of Phenolic Compounds from the Decoction of Ajuga iva *(L.)* by LC/UV/ESI-MS

**Figure 2:**
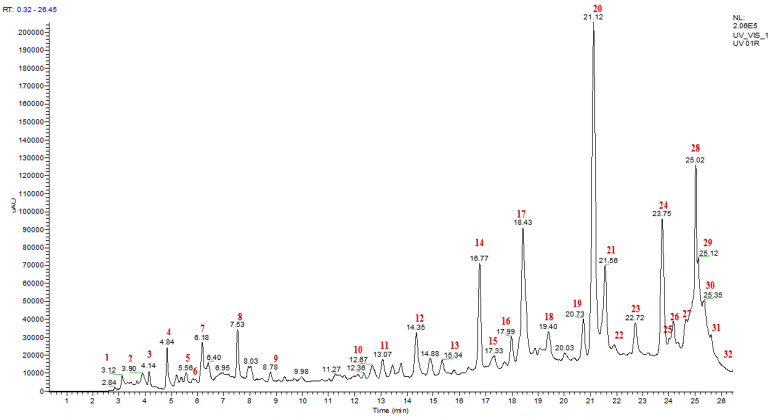
HPLC-MS chromatogram detected phenolic compounds from *Ajuga iva* (L.) decoction.

The analysis of the chemical composition of the polyphenols from *A. iva* (L.) leaves was performed by LC/UV/ESI-MS. The chromatographic profile presented below (Figure 2) illustrates the peaks of the constituents with their retention times and their relative abundance (%). Based on analytical and spectroscopic data (chromatogram and mass spectra), we detected and identified 37 compounds, whose names are presented in Table 7. All compounds were identified by interpreting their determined mass spectra and considering data reported in the literature. We have identified ten major compounds whose relative abundance is greater than 3%, these are ferulic acid (19.06%), quercetin (10.19%), coumaric acid (9.63%), apigenin-7-(2-O-apiosylglucoside) (6.8%), cholesterol (6.17%), luteolin (4.53%), ajugasterone D (4.29%), kaempferide (4.2%), epigallocatechin gallate (3.94%), and vanillin (3.17%). Ferulic acid, quercetin, and coumaric acid were the most abundant compounds found in the decoction of *A. iva* (L.). Various studies have shown the pharmacological properties of ferulic acid as an antioxidant [87,88], antiallergic, anti-inflammatory, hepatoprotective, anticancer, antimicrobial, antiviral, vasodilator, antithrombotic [89], and antidiabetic [90]. The same goes for the compound quercetin, which is known to have good anti-inflammatory, antioxidant [91], antidiabetic [92], anticancer, antiproliferative, apoptotic [93,94], antimicrobial, and antidiabetic effects [95]. In addition, coumaric acid also has antimicrobial, antioxidant, anticancer, immunoregulatory [96,97], antidiabetic, and antihyperlipidemic properties [98]. Moreover, new compounds called ajugasterone-7-(2-O-apiosylglucoside), ajugasterone D, and cholesterol have been discovered as a result of our research on the decoction of *A. iva* (L.). The presence of ferulic acid, which is a derivative of cinnamic acid, has been reported in a variety of studies that examined the chemical composition of the genus *Ajuga*. In addition, other bioactive compounds, such as p-coumaric acid, quercetin, and luteolin, were identified in these studies [99,100,101]. These results are similar to those of our study. Other chemical components found in our plant, such as epigallocatechin gallate, harpagide, and cyasterone, have also been reported in *A. iva* (L.) from Tunisia [66]. In conclusion, these results can confirm the use of *A. iva* (L.) leaves as natural alternatives to industrial products, especially in the pharmaceutical field.

### 3.6. Antioxidant Activity of Ajuga iva *(L.)* Extracts

The antioxidant activity of *A. iva* (L.) extracts is determined using three tests, DPPH, CAT, and FRAP. This antioxidant property for each extract is estimated by determining the 50% inhibitory concentration (IC_50_). The results of the antioxidant activity of *A. iva* (L.) depending on the extraction method used are shown in Table 8. The hydro-ethanolic extract showed the highest ability to scavenge free radicals of DPPH* (IC_50_ = 59.92 ± 0.70 µg/mL), followed by the aqueous extract prepared by Soxhlet (IC_50_ = 145.15 ± 0.72 µg/mL), and then the decoction that showed the lowest antioxidant activity (IC_50_ = 195.35 ± 14.58 µg /mL). The antioxidant activity of the extracts of this plant was also evaluated by testing their iron-reducing antioxidant power. Similarly, the hydro-ethanolic extract showed the highest reducing power (EC_50_ = 196.85 ± 1.54 µg/mL), followed by the aqueous extract prepared by soxhlet (EC_50_ = 290.04 ± 0.06 µg/mL), and then the decocted that showed the lowest reducing power (EC_50_ = 346.79 ± 3.59 µg/mL). For the total antioxidant capacity test, the hydro-ethanolic extract showed the highest total antioxidant capacity (199.21 ± 0.37 mg EAG/gE), followed by the aqueous extract prepared by Soxhlet (109.55 ± 2.24 mg EAG /gE), and then the decoction that showed the lowest total antioxidant capacity (117.33 ± 0.69 mg EAG/gE). The antioxidant activity of all these extracts remains lower in comparison with that of ascorbic acid used as a positive control (Table 8). The antioxidant activity of *A. iva* (L.) in this study is higher than the antioxidant activity of the same plant in other regions, such as *A. iva* (L.) from Oued Amlil region, Taza, Morocco [84], from the region from Bel-Abbass in northwestern Algeria [34], and *A. chamaecistus* from Taleqan region, Alborz province, Iran [102], using the DPPH test. This very significant antioxidant property of *A. iva* (L.) extracts is due to their richness in polyphenols and flavonoids. Indeed, several studies have reported that these secondary metabolites have very significant antioxidant activity [103,104]. Indeed, Makni and his collaborators worked on several extracts of *A. iva* (L.) using four solvents (methanol, chloroform, water, and hexane) and found that the methanolic and aqueous extracts that showed the best concentrations of phenolic compounds and flavonoids showed powerful results in terms of antioxidant capacity for all tests (DPPH*, FRAP, and CAT) [105]. This difference in the antioxidant activity of the three extracts studied is also mentioned in the work of Bouyahya et al. (2020), where they showed that the methanolic extract of *A. iva* (L.) has the highest antioxidant capacity compared to its aqueous extract [30].

### 3.7. Correlations between Antioxidant Activities and Phenolic Compound Contents of Ajuga iva *(L.)* Extracts

**Figure 3:**
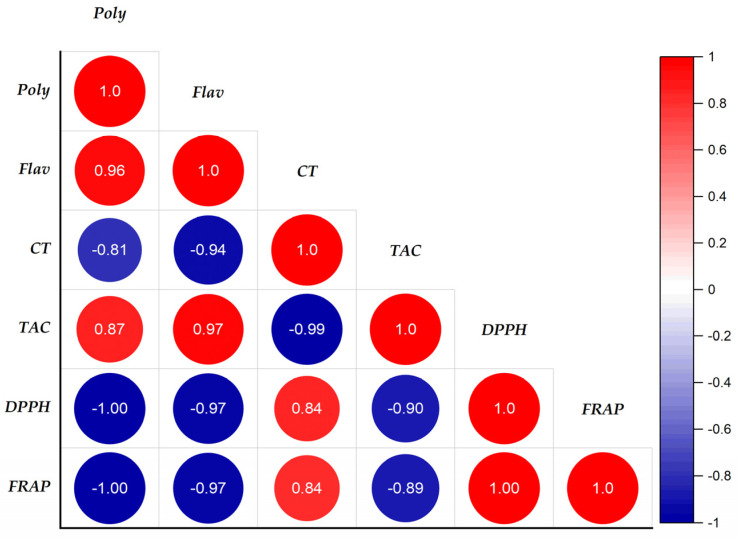
Correlation coefficient between the variables of polyphenolic compounds and biological activities in the extracts of the leaves of *Ajuga iva* (L.) from the region of Masmouda, Morocco.

The study of the correlation between the content of phenolics and the antioxidant activity of extracts of *A. iva* (L.) was established by determining the linear correlation coefficient (R^2^), known as the Bravais–Pearson coefficient. The results grouped together in Figure 3 show the correlation between the antioxidant activities (DPPH*, FRAP, and CAT) and the contents of phenolic compounds (polyphenols, flavonoids, and tannins).

We notice a strongly negative correlation between the contents of polyphenols, flavonoids, and the antioxidant activities DPPH* and FRAP, which gave coefficients of the order of −1 and −0.97, respectively. In addition, a strong positive correlation was observed between polyphenols, flavonoids, and total antioxidant activity with respective correlation coefficients R^2^ = 0.87 and R^2^ = 0.97. On the other hand, the tannins show no correlation with the antioxidant activities FRAP, DPPH*, and CAT, the correlation coefficients of which are as follows (R^2^_FRAP/Tannins_ = 0.84, R^2^_DPPH/Tannins_ = 0.84 and R^2^_CAT/Tannins_ = −0.99). From this, we deduced that the antioxidant power of the DPPH*, CAT, and FRAP tests of the *A. iva* (L.) extracts was due to the contribution of more than 87% of phenolic compounds and flavonoids, while the tannin content was very weak. In addition, there was a significant positive association between polyphenols and flavonoids (R^2^ = 0.98); on the other hand, a non-correlation was observed between catechin tannins with polyphenols and flavonoids. However, there was a strong positive correlation between the reductive power of iron (R^2^ = 1) and the scanning capability of DPPH* radicals.

The antioxidant activities of DPPH* and FRAP have a high negative correlation with the total antioxidant activity. R^2^ = −0.90 and R^2^ = −0.89, respectively, are the negative correlation coefficients of correlation. This indicates that in the extracts tested, the free radical scavenging power (DPPH*) and the iron reducing power (FRAP) are inversely proportional to the total antioxidant capacity. These results are in accordance with the data from the literature, through which several authors have shown the interdependence of potential antioxidant activities of an extract and the content of phenolic compounds [106,107,108].

### 3.8. Antimicrobial Activity of Ajuga iva Extracts

#### 3.8.1. Antifungal Activity

The antifungal activity of the three extracts (Eeth, EAq, and Ed) of *Ajuga iva* (L.) was evaluated against eight fungal strains *C. albicans*, *C. dubliniensis*, *S. cerevisiae*, *A. niger*, *C. tropicalis*, *C. krusei*, and *C. glabrata*, and these strains that are sensitive to the extracts were tested by the MIC and MFC methods. The findings are presented in Table 9.

The determination of the MIC of the extracts, with a range of concentration going from 12.5 to 100 mg/mL was not influenced either by the type of extraction (Soxhlet or decoction) or by the type of solvent (water and the ethanol–water mixture); the antifungal activity depended on the fungal species; the results obtained showed that the *C. parapsilosis* strain is the most sensitive to the three extracts of *A. iva* (L.); the MIC appears from the concentration of 6.25 mg/mL. The MICs recorded for the other strains varied depending on the type of extract, between 6.25 mg/mL to 100 mg/mL. Indeed, *C. dubliniensis*, *C. glabrata*, and *A. niger* resisted up to 25 mg/ mL for the hydroethanolic extract, whereas the strains *C. albicans*, *S. cerevisiae*, *A. niger*, *C. tropicalis*, *C. krusei*, and *C. glabrata* resisted up to 25 mg/ mL for the aqueous extract. Furthermore, *C. albicans S. cerevisiae*, *C. krusei*, and *C. glabrata* resisted up to 12.5 mg/mL for the decocted extract. The MFCs differ depending on the fungal species tested. *C. parapsilosis* was the most sensitive towards hydroethanolic, aqueous, and decocted extracts with a concentration of 6.25 mg/mL (Table 9).

*A. iva* (L.) has proven to have antibacterial characteristics in a number of investigations [31,32,34,105,109,110]. The Tunisian study on *A. iva* (L.) revealed that the methanolic extracts exhibited favorable antibacterial and antifungal activities [105]. This study tested three different fungal strains, *A. clavatus*, *A. niger*, and *Fusarium*, with *Fusarium* exhibiting the highest inhibitory activity and *A. niger* exhibiting the lowest inhibitory activity. The findings provide evidence that phenolic compounds of *A. iva* may be responsible for the antifungal/antimicrobial activities [111,112].

#### 3.8.2. Antibacterial Activity

According to the results of the antibacterial activity (Table 10), the MIC of the aqueous extract was revealed from a concentration of 3.13 mg/mL for *P. mirabilis* up to 100 mg/mL for *S. epidermidis* and *E. coli* BLSE for aqueous extract, while the hydroethanolic extract recorded MICs ranging from 12.25 mg/mL for *S. agalactiae* to 50 mg/mL for *S. epidermidis* and *P. aeruginosa*. Similarly, the decoction was more effective against *P. mirabilis* with a concentration of 12.5 mg/mL against the strains *P. mirabilis* and *S. aureus* BLACT to 100 mg/mL for *E. coli* BLSE. The lowest MBC values were recorded for *P. mirabilis* (12.5 mg/mL), while the highest were recorded for *E. coli* ESBL (100 mg/mL). We can then claim that *P. mirabilis* was the most sensitive, while *E. coli* ESBL was the most resistant. According to a study carried out by Bouyahya et al. [30], the antibacterial potential of hydroalcoholic and aqueous extracts by the MIC method was qualitatively evaluated against four Gram-positive strains and three Gram-negative strains. In fact, the aqueous extract showed to be slightly more effective than the extract hydroalcoholic against the growth of all tested bacteria. A significant amount of activity was shown against the *S. aureus* (MRSA) strain (80 < MIC < 155 μg/disk), and a considerable amount of activity was shown against the *S. aureus* and *B. cereus* strains (145 < MIC < 200 μg/disk). *L. monocytogenes* and all Gram-negative bacteria, on the other hand, were only faintly inhibited (625 < MIC < 1250 μg/disc) [34]. The results obtained demonstrate that there are differences between the effect of the extracts studied and the bacterial species. The synergistic interaction of all the chemical elements found in the extracts is what gives *A. iva* (L.) its strong antibacterial activity (Table 10). Characterized flavonoids may be the cause of *A. iva*’s powerful antibacterial activity. Different levels of these substances’ effects on microbial development are possible. Some flavonoids combine with extracellular proteins to create complexes and are thus only soluble with bacterial cell proteins [113]. These complexes influence the permeability of microbial membranes and alter the structure of their various layers of polysaccharides, fatty acids, and phospholipids [114]. The promising antibacterial and antifungal properties of *A. iva* (L.) according to our study, have demonstrated that certain compounds may be further evaluated as antimicrobial medicines for commercialization.

### 3.9. Acute Safety of the Decocted Extract of Ajuga iva *(L.)*

The decoction of this plant was not toxic even at 2 g/kg, according to the results of the acute toxicity test. Throughout the monitoring period, there were no symptoms of toxicity (diarrhoea, vomiting, altered movement, etc.) or mortalities. These results are exactly the same as those of Saad et al. [84]. They confirmed that the methanolic and aqueous extract had no short-term adverse effects in mice even at 2 g/kg [84]. The consumption of ivette by non-diabetics does not lead to a reduction in their blood sugar levels, whereas it has a hypoglycemic effect in people with diabetes [23]. By the oral route, the LD_50_ is greater than 14 g/kg BW, while that of the intraperitoneal route is approximately 3.6 g/kg BW. As for the chronic treatment (up to 600 mg/kg BW), it does not have any harmful effects on the biochemical and hematological parameters. A histological analysis of vital organs reflects normal anatomorphological structures [115], and the LD_50_ shows that the flavonoids of *A. iva* (L.) are not toxic due to their high values (3600 mg/kg for mice and 4800 mg/kg for rats) [116].

### 3.10. Antihyperglycemic Effect of Decocted Extract of Ajuga iva *(L.)*

**Figure 4:**
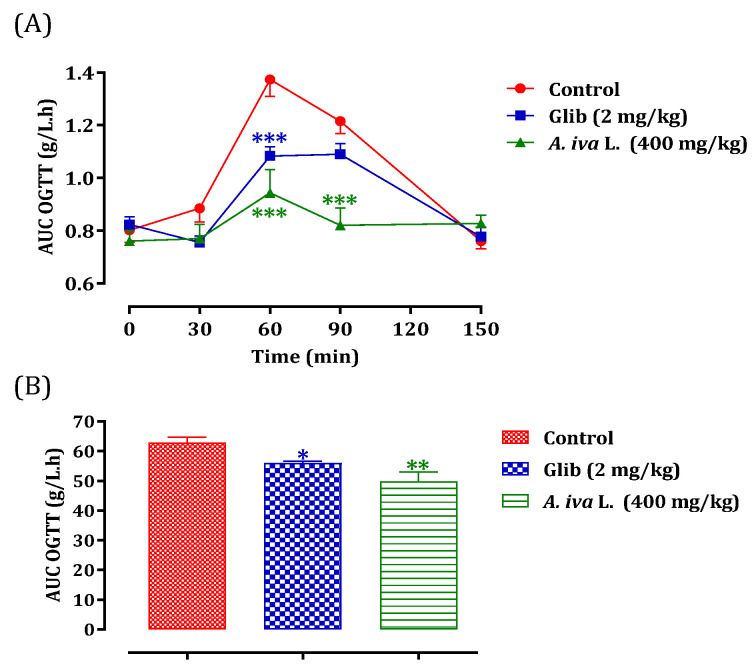
Postprandial glycaemia variation (**A**) and AUC (**B**) in normal rats after administration of *A. iva* L. decoction and glibenclamide. Values are SEM means. (*n* = 6). *** *p* < 0.001; ** *p* < 0.01; * *p* < 0.05: compared to the control.

The antihyperglycemic effect is tested for the decoction of *A. iva* (L.) leaves. The choice of plant material and extraction technique among the other two mentioned above is based on the traditional use of this plant in traditional medicine [30]. Oral administration of *A. iva* (L.) decoction (40 mg/mL) made it possible to significantly reduce postprandial hyperglycemia in normal rats at 60 (*p* < 0.001, 0.94 ± 0.22 g/L) and 90 min (*p* < 0.01, 0.82 ± 0.16 g/L). In the same way, glibenclamide reduced—significantly—postprandial hyperglycemia at 60 min (*p* < 0.001; 1.08 ± 0.09 g/L) and 90 min (*p* < 0.05; 1.09 ± 0.10 g/L). At 150 min, no significant difference in glycaemia values was observed in either group, compared to the distilled water pretreated group (at 60 min (1.37 ± 0.16 g/L) at 90 min (1.22 ± 0.11 g/L) and at 150 min (0.76 ± 0.07 g/L)) (Figure 4). The AUC was also substantially lower (*p* < 0.01) in rats administered with the aqueous extract of *A. iva* (L.) (49.81 ± 7.61 g/L/h) than the distilled-water-treated ones (62.91 ± 4.32 g/L/h). Additionally, glibenclamide’s AUC was considerably (*p* < 0.01) lower (55.95 ± 1.69 g/L/h) than the AUC of rats given distilled water (62.91 ± 4.32 g/L/h) (Figure 4). According to our knowledge, many studies have generally assessed the hypoglycemic effect of this plant in different animal models of diabetes [29,30,84,117,118], while few studies that have been interested in the mechanism of action of this study. Therefore, in this study we tried to test whether this already proven anti-diabetic effect could be due to its postprandial anti-hyperglycemic ability, using the oral glucose tolerance test. As we mentioned in the results of this test, the antidiabetic effect of this plant is due to its property of reducing the increase in blood sugar caused by the overload of foods rich in carbohydrates. A phytochemical analysis of the aqueous extract of the aerial part of *A. iva* (L.) showed its richness in apigenin (flavonoid compound) [29]; this flavonoid molecule showed a hypoglycemic effect in diabetic rats by reducing glucose absorption, stimulating insulin secretion, and stimulating glycogen synthesis in the muscles. This finding implies that this flavonoid may have two opposing effects by promoting both glycogen production and insulin secretion (antihyperglycemic) (insulin mimetic) [119,120,121]. Additionally, a phytochemical analysis of the aerial section of *Ajuga iva* found that an essential steroid component called ecdysterone was present in the aqueous extract. This compound has the ability to potentiate the utilization of glucose by cells [122]. In fact, regardless of the concentration of insulin present and without causing any adverse effects, this steroid had this impact on hepatocytes [123].

### 3.11. Inhibitory Effect of Decocted Extract of Ajuga iva *(L.)* on Pancreatic α-Amylase

#### 3.11.1. In Vitro Test

**Figure 5:**
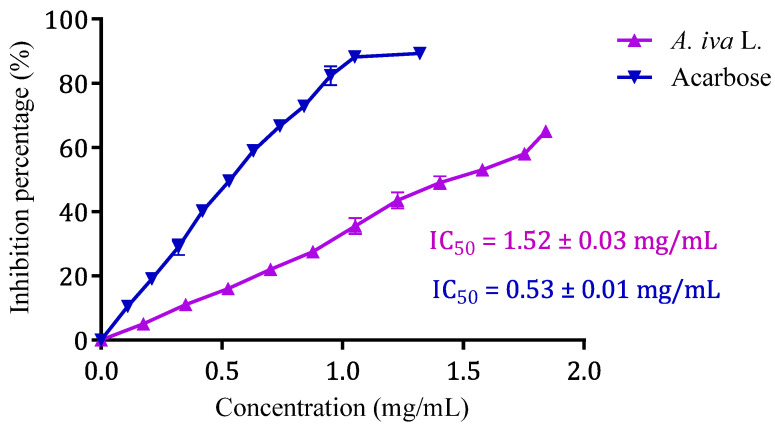
*Ajuga iva* (L.) aqueous extract and acarbose inhibitory effect on α-amylase activity in vitro. Values are means ± SEM, (*n* = 3).

Glucose is produced during the catabolism of complex carbohydrates, such as starch, and its absorption raises postprandial glycaemia. Therefore, the inhibition of enzymes that mediate the intestinal digestion of these carbohydrates is an important strategy for controlling blood sugar in diabetics [30]. Acarbose is a drug that inhibits the enzymatic activity of the brush border, maltase, sucrase, glucoamylase, dextrinase, as well as pancreatic α-amylase [124]. Despite the use of this molecule as an antidiabetic agent, an alternative plant-based antidiabetic product is highly essential. Additionally, long-term consumption of acarbose has caused side effects and rare cases of hepatotoxicity [125]. This is why scientists are interested in functional foods and nutritional therapies with curative and preventive effects against diabetes and obesity [126]. The results of the effect of *A. iva* (L.) decoction on the in vitro pancreatic α-amylase activity are illustrated in Figure 5. Indeed, pancreatic α-amylase activity was significantly inhibited by the aqueous extract of *A. iva* with an IC_50_ of 1.52 ± 0.03 mg/mL. Moreover, this inhibitory property on the enzymatic activity of pancreatic α-amylase was superior to that of acarbose (IC_50_ = 0.53 ± 0.01 mg/mL). As far as we are aware, not many studies have looked into how this herb affects pancreatic α-amylase. In fact, the inhibitory effect of our aqueous extract on pancreatic α -amylase activity is substantially lower than that seen by Fettach and colleagues for an aqueous extract of the same plant in the region of Oued Amlil, Taza, Morocco (IC_50_ = 0.21 ± 0.00 mg/mL) [84], and it is higher than the value found by Senhaji and his collaborators for the same plant in the Taza region (2.21 ± 0.24 mg/mL).

#### 3.11.2. In Vivo Test

**Figure 6:**
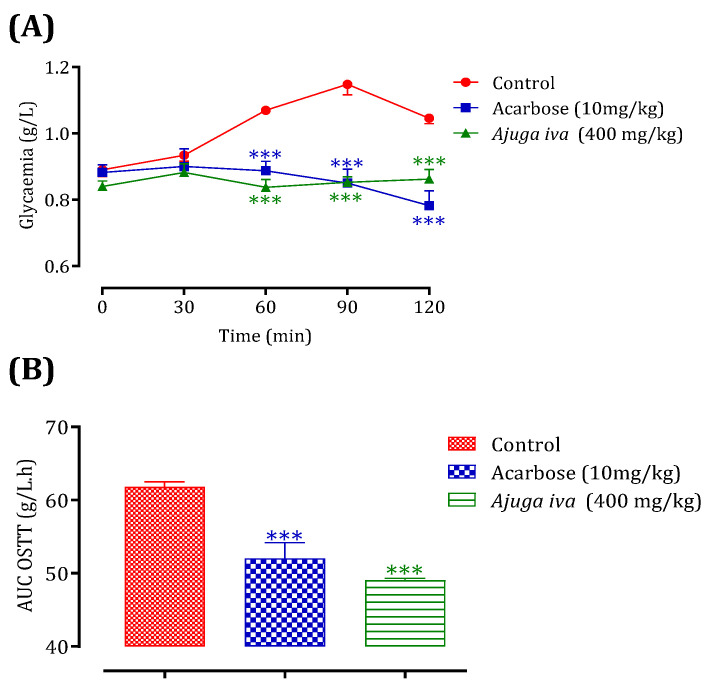
*Ajuga iva* (L.) and acarbose effect on the variation of postprandial glycaemia in normal rats (**A**), with a representation in the form of areas under curves (**B**). Values are SEM means. (*n* = 6). *** *p* < 0.001: compared to control.

This test was validated in vivo using the Bouhrim et al. [127] approach to account for the impact of the intestinal environment on the inhibitory action of this plant on the α-amylase enzyme. To the best of our knowledge, no studies have been performed regarding the inhibitory effect of *A. iva* (L.) decoction on α-amylase in vivo. For this reason, the oral administration of *A. iva* (L.) decoction, with a concentration of 40 mg/mL, 30 min before starch overload in our normal rats allowed a significant reduction in their postprandial hyperglycemia at 60, 90, and 120 min (*p* < 0.001), with the respective values of 0.84 ± 0.05 g/L, 0.85 ± 0.03 g/L and 0.86 ± 0.06 g/L compared to the group pretreated with distilled water. The latter, when overloaded with starch, induced remarkable hyperglycemia at t = 60 min (1.07 ± 0.02 g/L), 90 min (1.15 ± 0.07 g/L), and 120 min (1.05 ± 0.04 g/L) [56]. On the other hand, acarbose significantly reduced postprandial hyperglycemia at 60 min (*p* < 0.001, 0.89 ± 0.06 g/L), 90 min (*p* < 0.001, 0.85 ± 0.08 g/L), and 120 min (*p* < 0.001, 0.78 ± 0.09 g/L) over the 2 h after starch excess (Figure 6A). Additionally, rats fed with the decoction had an area under curve (AUC) that was considerably lower (*p* < 0.001) than rats treated with distilled water (61.82 ± 1.53 g/L/h). Additionally, the area under the curve for acarbose was substantially (*p* < 0.001) lower (52.05 ± 4.27 g/L/h) than it was for rats administered with water (61.82 ± 1.53 g/L/h) (Figure 6B). The inhibitory effectiveness of this plant extract in comparison to acarbose is confirmed by this in vivo test.

From these results, it is clear that the *A. iva* (L.) leaves could be used to treat postprandial hyperglycemia in diabetics. On the one hand, the aqueous extract is rich in natural bioactive molecules (apeginen 7-O-glucoside, ferulic acid, coumaric acid, quercetin, etc.) in addition to nutrients. On the other hand, the ease of their administration and the availability and convenient cost of the plant make this plant a potential competitor to the synthetic drug ‘acarbose’.

## 4. Conclusions

In this work, chemical, biological, and pharmacological studies were carried out on the leaves of *A. iva* (L.). The results obtained demonstrate a positive correlation between the biological activities of the extracts studied and the presence of phenolic compounds. Thus, the chemical composition and the richness in minerals of our plant prove the important therapeutic effect of *A. iva* (L.). The antidiabetic activity carried out on the decoction, the most commonly used extract in traditional medicine, revealed a good inhibition of the activity of the enzyme α-amylase, thus decreasing postprandial hyperglycemia. The results observed in the decoction extract could be attributed to the existence of polyphenolic compounds, including ferulic acid, quercetin, coumaric acid, luteolin, etc.

Given these findings, the antidiabetic, antioxidant, and antimicrobial qualities identified during this study validate and support the Moroccan population’s traditional usage of *A. iva* (L.) leaves as a natural antidiabetic and/or antimicrobial remedy.

## Figures and Tables

**Table 1:** *Ajuga*’s taxonomic position in the family of plants [36].

Reign	Plantae
Division	Spermatophyta
Class	Dicotyledones
Order	Tubiflorae
Family	Lamiaceae
genus	*Ajuga*
Species	*Iva*
Author	(L.) Schreber

**Table 2:** List of bacterial and fungal strains tested.

Bacterial Strains	Reference	Fungal Strains	Reference
*Staphylococcus epidermidis*	5994	*Candida albicans*	Ca
*Staphylococcus aureus* BLACT	4IH2510	*Candida dubliniensis*	Cd
*Streptococcus agalactiae* (B)	7DT1887	*Saccharomyces cerevisiae*	Sacc
*Escherichia coli sauvage*	3DT1938	*Aspergillus niger*	AspN
*Escherichia coli* BLSE	2DT2057	*Candida tropicalis*	Ct
*Enterobacter cloacae*	02EV317	*Candida krusei*	Ckr
*Klebsiella pneumoniae*	3DT1823	*Candida parapsilosis*	Cpa
*Proteus mirabilis*	2DS5461	*Candida kyfer*	C.ky
*Pseudomonas aeruginosa*	2DT2138		

**Table 3:** Parameters evaluated of plant material from *Ajuga iva* (L.).

Plant	Moisture Content (%)	pH	Ash (%)	Titratable Acidity (%)
*A. iva*	11.56 ± 0.16	5.28 ± 0.10	16.30 ± 0.28	37.87 ± 0.01

**Table 4:** Heavy metal content (mg/g) (ICP) of plant material from *Ajuga iva* (L.).

*Ajuga iva* (L.)	As	Cr	Sb	Pb	Cd	Fe	Cu	Ti
Content (mg/g)	0.0213	0.0297	0.0239	0.0243	0.0015	2.3433	0.0082	0.0257

**Table 5:** Phytochemical screening of *Ajuga iva* (L.) leaves.

Chemical Groups			Observations
Primary metabolites	Polysaccharides		*+* Glycogen
	Lipids		+++
	Proteins	R.Biuret	+
		R.Xantho	++
	Reducing sugars		+
Secondary metabolites	Tannins	Gallic	++
		catechists	++
	Flavonoids	Flavones	++
		Leucoanthocyanins	++
	Anthocyanins		++
	Saponosides		−
	Alkaloids	Mayer	+
		Dragendorff	+
		Wagner	++
	Reducing compounds		+++
	Monosaccharides and holosides		+++
	Mucilages		−
	Sterols and triterpenes		++

+: weak positive test; ++: positive test; +++: strongly positive test; −: negative test.

**Table 6:** Extraction yields and phenolic compound contents of *Ajuga iva* (L.) extracts.

Extract	Extraction Yields (%)	Polyphenols (mg EAG/g E)	Flavonoids (mg EQ/g E)	Catechin Tannins (mg EQC/g E)
Decocted	17.016 ± 0.032	40.786 ± 2.957	8.960 ± 0.370	5.746 ± 0.413
Aqueous extract	11.048 ± 0.511	53.148 ± 2.509	10.281 ± 0.521	5.792 ± 0.064
Hydroethanolic extract	7.385 ± 0.662	69.850 ± 2.783	17.127 ± 0.474	5.566 ± 0.000

**Table 7:** Chemical composition of *Ajuga iva* (L.) decoction by LC/UV/MS.

N°	TR (min)	A.R. (%)	[M − H]^−^ (*m*/*z*)	[M + H]^+^ (*m*/*z*)	MW	Compounds Identified
1	3.12	0.43	195	-	196	Galacturonic acid
2	3.9	0.79	317	-	318	Myricetin
3	4.14	0.73	169	-	170	Gallic acid
4	4.84	1.37	175	-	176	Ascorbic acid
5	5.56	1.3	209	-	210	Mucic acid
6	5.82	0.33	173	-	174	Arginine
7	6.18	3.17	151	-	152	Vanillin
8	7.53	2.01	191	-	192	Quinic acid
9	8.78	1.06	147	-	148	Cinnamic acid
10	12.67	1.3	315	-	316	Rhamnetin
11	13.07	2.04	451	-	452	Catechin-7-O-glucoside
12	14.35	2.43	289	-	290	Catechin
13	15.34	1.39	363	-	364	Harpagid
14	16.77	4.29	477	-	478	Ajugasterone D
15	17.33	1.41	145	-	146	Coumarin
16	17.99	1.26	-	357	356	Ferulic acid 4-O-glucoside
17	18.43	9.63	163	-	164	Coumaric acid
18	19.4	3.94	-	459	458	Epigallocatechin gallate
19	20.73	1.96	405	-	406	8-O-acetyl-harpagid
20	21.12	19.06	193	-	194	Ferulic acid
21	21.56	6.17	385	-	386	Cholesterol
22	21.9	0.77	519	-	520	Cyasterone
23	22.72	1.88	389	-	390	Resveratrol 3-Glucoside
24	23.75	6.8	563	-	564	Apigenin-7-(2-O-apiosylglucoside)
25	24.03	0.43	-	651	650	Apigenin 7-O-(6″-malonyl-apiosyl-glucoside)
26	24.17	1.38	269	-	270	Apigenin
27	24.65	1.45	565	-	566	Quercetin-3-O-pentosyl-pentoside
28	25.02	10.19	301	-	302	Quercetin
29	25.12	4.25	299	-	300	Kaempferide
30	25.35	4.53	387	-	388	Luteolin
31	25.61	1.87	-	327	326	Trans-p-coumaric acid
32	26.49	0.31	329	-	330	Vanillic acid glucoside
Total: 99.93%						

**Table 8:** Antioxidant activities of *Ajuga iva* (L.) extracts (by different methods).

Extracts	DPPH* IC_50_ (µg/mL)	CAT (mg EAG/gE)	FRAP EC_50_(µg/mL)
Decocted	195.35 ± 14.58	117.33 ± 0.69	346.79 ± 3.59
Aqueous extract	145.15 ± 0.72	109.55 ± 2.24	290.04 ± 0.06
Hydro-ethanolic extract	59.92 ± 0.70	199.21 ± 0.37	196.85 ± 1.54
Ascorbic acid	3.23 ± 0.11	-	9.27 ± 0.00

**Table 9:** MIC and MFC values for the examined microbial strains.

Microorganism	MIC Extracts (mg/mL)					
	Decocted		Aqueous		Hydroethanolic	
	MIC	MFC	MIC	MFC	MIC	MFC
*Candida albicans*	12.5	50.0	25.0	25.0	12.5	25.0
*Candida dubliniensis*	25.0	25.0	50.0	50.0	25.0	25.0
*Saccharomyces cerevisiae*	12.5	25.0	25.0	25.0	50.0	50.0
*Aspergillus niger*	50.0	50.0	25.0	25.0	25.0	25.0
*Candida tropicalis*	50.0	50.0	25.0	25.0	50.0	50.0
*Candida krusei*	12.5	25.0	25.0	25.0	50.0	100.0
*Candida glabrata*	12.5	25.0	25.0	25.0	25.0	50.0
*Candida parapsilosis*	6.25	12.5	6.25	12.5	6.25	12.5

**Table 10:** MIC and MBC values for the examined microbial strains.

Bacteria	Extracts (mg/mL)					
	Decocted		Aqueous Extract		Hydroethanolic	
	MIC	MBC	MIC	MBC	MIC	MBC
*Staphylococcus epidermidis*	50.0	100.0	100.0	50.0	50.0	100.0
*Staphylococcus aureus* BLACT	12.25	50.0	12.25	50.0	25.0	25.0
*Streptococcus agalactiae* (B)	50.0	100.0	25.0	25.0	12.25	25.0
*Escherichia coli* sauvage	50.0	50.0	25.0	25.0	25.0	25.0
*Escherichia coli* BLSE	100.0	100.0	100.0	100.0	25.0	50.0
*Entérobactérie cloacae*	50.0	100.0	25.0	25.0	25.0	25.0
*Klebsiella pneumoniae*	50.0	50.0	25.0	50.0	25.0	50.0
*Proteus mirabilis*	12.5	25.0	3.13	12.5	25.0	25.0
*Pseudomonas aeruginosa*	50.0	50.0	50.0	50.0	50.0	50.0

## Data Availability

Not applicable.
